# Structure of an Odorant-Binding Protein from the Mosquito *Aedes aegypti* Suggests a Binding Pocket Covered by a pH-Sensitive “Lid”

**DOI:** 10.1371/journal.pone.0008006

**Published:** 2009-11-26

**Authors:** Ney Ribeiro Leite, Renata Krogh, Wei Xu, Yuko Ishida, Jorge Iulek, Walter S. Leal, Glaucius Oliva

**Affiliations:** 1 Centro de Biotecnologia Molecular Estrutural, Instituto de Física de São Carlos, Universidade de São Paulo, São Carlos, São Paulo, Brazil; 2 Department of Entomology, University of California Davis, Davis, California, United States of America; 3 Departamento de Química, Universidade Estadual de Ponta Grossa, Ponta Grossa, Paraná, Brazil; Universidade Federal do Rio de Janeiro, Brazil

## Abstract

**Background:**

The yellow fever mosquito, *Aedes aegypti*, is the primary vector for the viruses that cause yellow fever, mostly in tropical regions of Africa and in parts of South America, and human dengue, which infects 100 million people yearly in the tropics and subtropics. A better understanding of the structural biology of olfactory proteins may pave the way for the development of environmentally-friendly mosquito attractants and repellents, which may ultimately contribute to reduction of mosquito biting and disease transmission.

**Methodology:**

Previously, we isolated and cloned a major, female-enriched odorant-binding protein (OBP) from the yellow fever mosquito, AaegOBP1, which was later inadvertently renamed AaegOBP39. We prepared recombinant samples of AaegOBP1 by using an expression system that allows proper formation of disulfide bridges and generates functional OBPs, which are indistinguishable from native OBPs. We crystallized AaegOBP1 and determined its three-dimensional structure at 1.85 Å resolution by molecular replacement based on the structure of the malaria mosquito OBP, AgamOBP1, the only mosquito OBP structure known to date.

**Conclusion:**

The structure of AaegOBP1 ( = AaegOBP39) shares the common fold of insect OBPs with six α-helices knitted by three disulfide bonds. A long molecule of polyethylene glycol (PEG) was built into the electron-density maps identified in a long tunnel formed by a crystallographic dimer of AaegOBP1. Circular dichroism analysis indicated that delipidated AaegOBP1 undergoes a pH-dependent conformational change, which may lead to release of odorant at low pH (as in the environment in the vicinity of odorant receptors). A C-terminal loop covers the binding cavity and this “lid” may be opened by disruption of an array of acid-labile hydrogen bonds thus explaining reduced or no binding affinity at low pH.

## Introduction

Most insect species are reliant on chemical communication to locate friends and foes, food sources, oviposition sites, etc. Female moths (in the order Lepidoptera) advertize their readiness to mate by releasing sex pheromones, which are utilized by male moths as trails to females. Mosquitoes (order Diptera), on the other hand, use airborne chemical signals (semiochemicals) to locate and determine suitability of hosts for blood feeding and sites for oviposition. Chemical communication plays such a pivotal role in insect's life that it can be manipulated with synthetic semiochemicals for trapping insect for surveillance and monitoring population levels as well as for management of populations for reduction of disease transmission and crop damage.

Odorant-binding proteins (OBPs; aka PBPs, pheromone-binding proteins when they are involved in the detection of pheromones) are the first relay in semiochemical reception in insects as they are the liaison between the air medium that broadcasts chemical signals and odorant receptors, which are located in olfactory structures (mainly the antennae and maxillary palps) of the insect's peripheral sensory system. These proteins, first isolated from moths [1], may serve as molecular targets for the development of attractants for mosquitoes [2], [3], moths [4], and other insect species. This reverse chemical ecology approach [5] relies on the affinity of test ligands to OBPs, which could be optimized by determining OBPs structural features. Prior to unveiling three-dimensional structures of insect OBPs, we observed that the PBP from the silkworm moth, BmorPBP-1 [6], undergoes a pH-dependent conformational change [7] implicated in loss of binding affinity at low pH [7], [8], [9]. Structural studies showed that an extended C-terminus of this protein [10], [11] forms an extra α-helix at low pH [10], which competes with the pheromone ligand. This pH-dependent mechanism for pheromone binding and release has been demonstrated in a number of lepidopteran PBPs [4], [7], [12], [13]. As highlighted by various species of mosquitoes (reviewed in [14]), dipteran OBPs are shorter (≈125-amino-acid-residues) than moth OBPs (≈140-amino-acid-residues) thus lacking the extended C-terminus to take over the binding pocket at low pH. Contrary to lepidopteran OBPs for which multiple structures have been determined [10], [11], [15], [16], [17], [18], [19], the structure of only one mosquito OBP, the malaria mosquito *Anopheles gambiae* AgamOBP1, has been reported to date [20]. Previously, we suggested that lowering pH may disrupt hydrogen bonds in AgamOBP1 and expose the binding cavity as the C-terminus forms a wall over the binding pocket by hydrophobic and polar contacts with the N-terminus and surrounding helices [20]. Recently, it was proposed that an OBP from the European honeybee, *Apis mellifera* (order Hymenoptera), undergoes a pH-induced domain swapping [21] raising the question whether “short” OBPs have two pH-mediated modes of action. We studied the structure of the major, female-enriched odorant binding protein (AaegOBP1) from *Aedes aegypti*, the primary vector for the viruses that cause yellow fever, mostly in tropical regions of Africa and in parts of South America, and human dengue, which infects 100 million people yearly in the tropics and subtropics. This “short” OBP, which was first isolated by us [22], but later inadvertently named AaegOBP39 [23], [24], showed similar pH-labile interactions as AgamOBP1, which seems to be a common feature of mosquito OBPs.

## Results and Discussion

### Overall Structure

Mature AaegOBP1 is a protein with 125 amino acid residues [22], which shares 82% amino acid identity (91% similarity) with AgamOBP1 [25]. One clear molecular replacement solution for the crystal asymmetric unit was found with PHASER with Z-score 35.1 and R_factor_ 38%, which consists of two monomers in the asymmetric unit, corresponding to Matthews coefficient [26] of 1.95 Å^3^ Da^−1^ and solvent content of ca. 37%. Electron density was well defined for most of the structure, except for a few side chains, the first residue of chain A, and the nine initial residues of chain B. The final refined structure contains two monomers of AaegOBP1 with 240 residues, 410 water molecules, 3 Mg^2+^ ions, one Cl^−^ ion and one putative PEG molecule. The final *R_factor_* and *R_free_* values were 0.151 and 0.212, respectively. The quality of the model, as analyzed by PROCHECK [27], shows that 93.4% of the residues are in the most favored region and the rest are in the additionally allowed region of the Ramachandran plots [28]. Complete refinement statistics are given in Table 1.

**Table 1:** Summary of data-processing and refinement statistics.

Wavelength/Å	0.9000
Space group	P2_1_
Overall resolution range/Å	70.0–1.85 Å
Unit-cell parameters/Å,°	a = 34.25, b = 47.87, c = 69.08, β = 96.61
No. of observed/unique reflections	139219/19077
Refined mosaicity/°	0.312
Completeness of data (%)	99.6 (98.9)
Redundancy	7.0 (7.4)
*R*_symm_ (%)	7.1 (54.7)
<*I*/(*I*)>	20.4 (4.4)
*R*_factor_ (%)	15.1
*R*_free_ (%)	21.2
Wilson *B* factor/Å^2^	21.4
R.m.s.d. from ideal values, bond lengths (Å)	0.010
R.m.s.d. from ideal values, bond angles/°	1.188
Overall *B* factors/Å^2^	
Protein atoms	25.1
Water molecules	27.8
Ions	32.3
PEG molecule	40.9
Ramachandran plot analysis	
Residues in most favored regions (%)	93.8
Residues in additional allowed regions (%)	6.2

Values in parentheses are for the highest resolution bin (1.92–1.85 Å)

**Figure 1:**
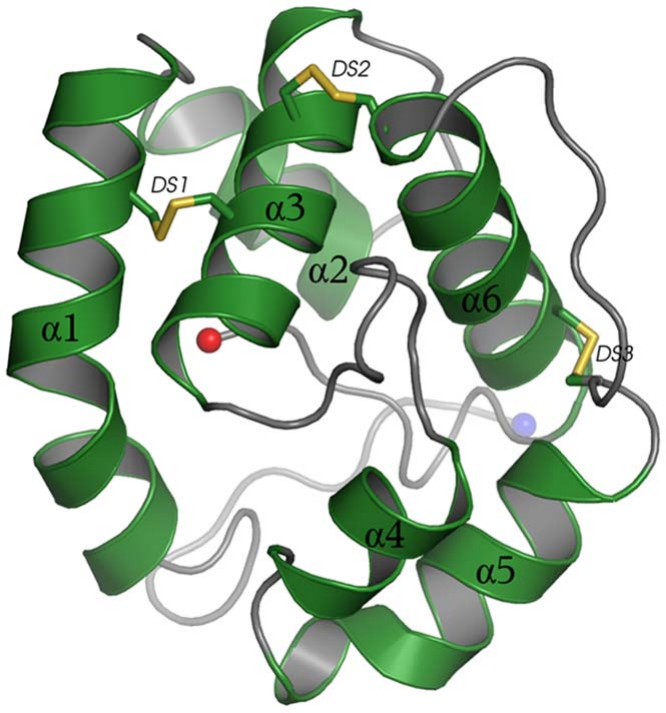
Cartoon representation of AaegOBP1 structure. α-Helices are shown in green, loops in gray and three disulfide linkages (DS1-3) knitting α-helices are highlighted in yellow. N- and C-terminus residues are denoted with blue and red spheres, respectively.

The overall structure of AaegOBP1 comprises of six helices (α1 to α6) connected by loops between helices and knitted together by three disulfide bridges between α1 and α3 (C26–C57), helix α3 and the top of helix α6 (C53–C104), and helix α6 and the top of helix α5 (C113–C95) (Fig. 1). Several map calculations indicated that residue Cys-53 of monomer A is better modeled as partially reduced, probably due to radiation damage. The hydrophobic residues Phe15, Leu58, Phe59, Ala62, Val64, Leu73, Leu76, Ala79, Leu80, Ala88, Leu89, Gly92, Leu 96, Phe123, Leu124 and Ile125 from helices 1, 3, 4, 5, and loops between helices 3 and 4, and 5 and 6 form the binding cavity. Interestingly, the C-terminus is pulled to the core of the protein to form part of the binding pocket wall, which can function as a “lid” for the release of ligands. The overall fold of six helices knitted together by three disulfide bridges and containing a hydrophobic binding cavity has been observed in other OBPs [11], [15], [18], [19], [20], [21], [29], [30], [31], but the C-terminal “lid” is unique to mosquito OBPs [20].

### Structural Comparisons

**Figure 2:**
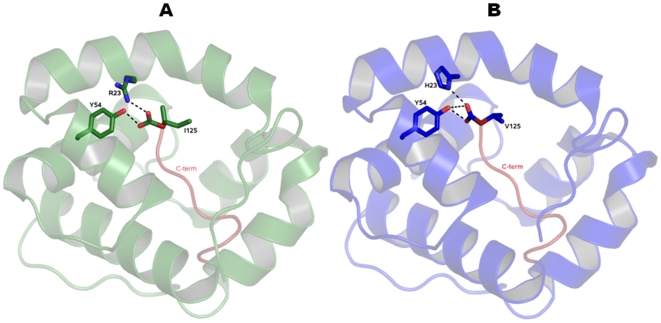
Comparison of AaegOBP1 and AgamOBP1 structures. Hydrogen bonding between the hydroxyl of Tyr-54 and the C-terminal carboxylate of Val-125 and Ile-125 from (A) AaegOBP1 and (B) AgamOBP1, respectively. The terminal oxygen makes hydrogen bonds with δ-nitrogen of His-23 (A) and Arg-23 (B), respectively.

**Figure 3:**
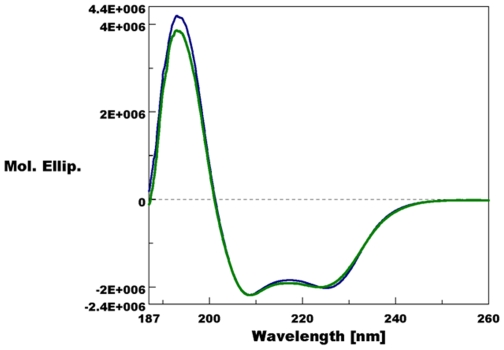
Circular dichroism data. Far-UV CD spectra of AaegOBP1 at the postulated sensillar lymph pH 7 (blue trace) and at pH 5.5 (green trace). Except for a small change in the second minima, the two traces are almost indistinguishable, thus suggesting no change in the overall secondary structure at low pH.

**Figure 4:**
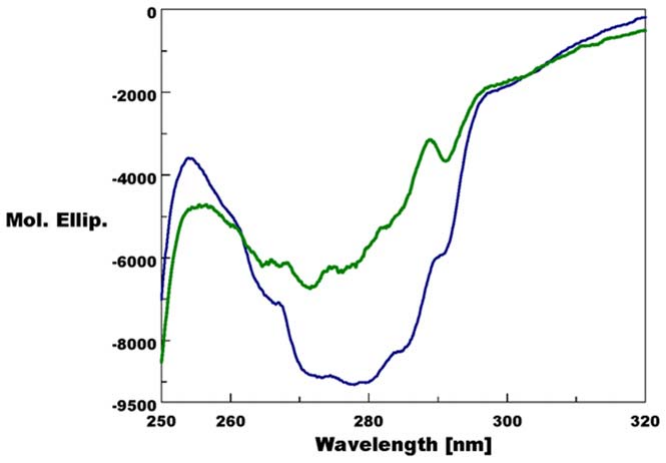
Circular dichroism data. Near-UV CD spectra of AaegOBP1 at pH 7 (blue trace) and pH 5.5 (green trace). Disruption of hydrogen bonds that keep the C-terminus covering the binding pocket may account for the changes in the environment of aromatic residues (Phe, Tyr, and/or Trp) at low pH and consequently the decrease in amplitude.

Structural superpositions between the refined main chain atoms of AaegOBP1 and AgamOBP1 give RMSD values ranging from 0.29 to 0.40 Å depending on the pair of chains superposed, which is not entirely surprising given the high amino acid identity (82%). The C-terminal “lid” is held in place by an array of hydrogen bonds, which is well-conserved between AgamOBP1 [20] and AaegOBP1, namely: Arg5-Asp7, Arg6-Asp42, His121-Asp118, Asp118-Lys120 and Tyr10-Asp7. The C-terminal “lids” in AgamOBP1 and AaegOBP1 differ only in the last residue: Val-125 vs. Ile-125, respectively. The carboxylate oxygens of these residues form hydrogen bonds with the hydroxyl of Tyr-54 and the δ-nitrogen of His-23 (AgamOBP1) (Fig. 2A) and Arg-23 (AaegOBP1) (Fig. 2B). It is likely that some of these interactions may be disrupted at low pH [20] leading the C-terminus to move away from the binding cavity thus “opening of the lid” and loosing ligand affinity at low pH. Indeed, AaegOBP1 binds to a mosquito attractant, nonanal [2], with apparent high affinity at the estimated pH of the sensillar lymph [32], but with no affinity at the low pH postulated for the environment in the vicinity of odorant receptors due to negative charges surfaces in the neuron membrane [33], [34]. We examined by circular dichroism (CD) the effect of lowering pH on the secondary and tertiary structures of AaegOBP1. As expected, the far-UV CD spectra for AaegOBP1 at high- and low-pH were almost indistinguishable (Fig. 3), with a maximum at 193 nm and two minima, one at 209 nm and the other at 224 (at pH 5.5) and 225 nm (pH 7). These spectral data suggest that lowering the pH did not disrupt the overall secondary structure of the protein. The near-UV CD spectra, on the other hand, showed a remarkable change in the tertiary structure by lowering the pH (Fig. 4). The disruption of hydrogen bonds holding the C-terminal loop in AaegOBP1 would cause this binding pocket cover to move away and, consequently, expose Tyr, Trp and Phe residues, particularly Phe-123, Trp-114, and Tyr-122 in the C-terminus, to different environments; Phe-108 and Trp-109 in α-helix 6 may be affected, too.

**Figure 5:**
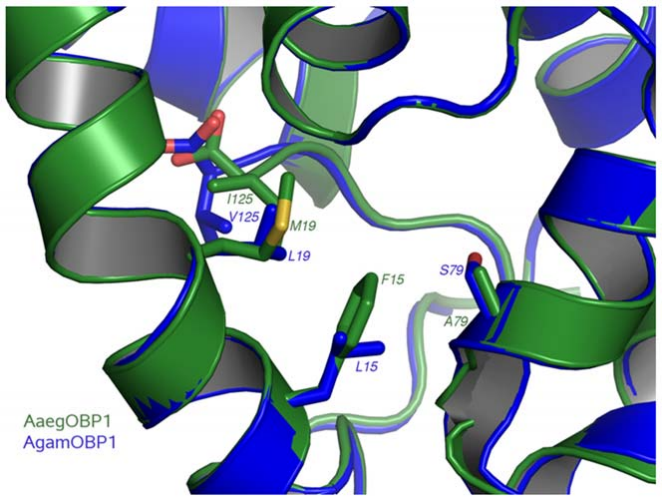
Superposition of the binding pockets of AaegOBP1 and AgamOBP1. Clusters of different residues in the binding pockets of AaegOBP1 (green) and AgamOBP1 (blue) are highlighted. These residues may confer ligand binding specificity between these two OBPs.

**Figure 6:**
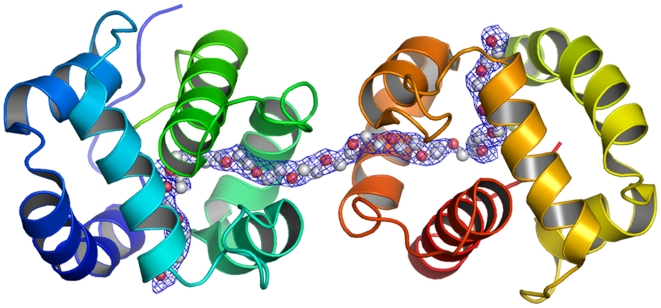
AaegOBP1 binding pocket highlighting a tunnel running through two units of a crystallographic dimer. One PEG molecule (carbon, light gray; oxygen, red) could be modelled in an ordered fashion to a stretch of 55 atoms.

**Figure 7:**
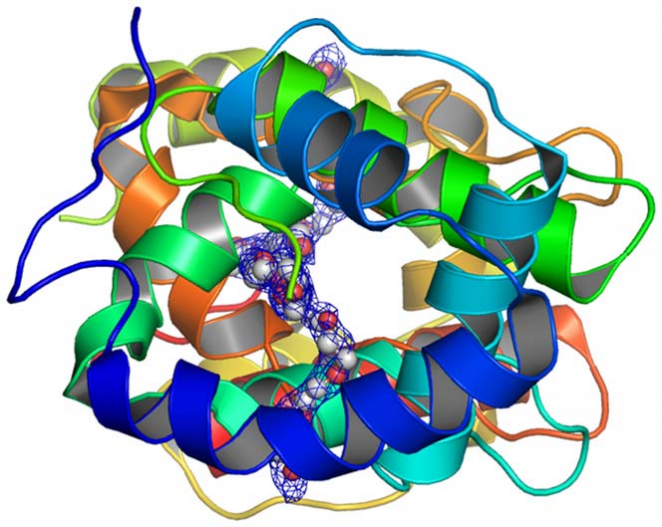
AaegOBP1 binding pocket with a PEG molecule. The structure in Fig. 6 was rotated by 90°.

Differences in ligand specificities by these two proteins might be conferred by different residues in the binding cavity, which are located close to the C-terminal lids in AgamOBP1 and AaegOBP1: Leu-15 vs. Phe-15, Leu-19 vs. Met-19, and Ser-79 vs. Ala-79 (Fig. 5). The binding cavities of the two proteins have two entrances, separated from each other by helix 3, and forming one channel from one side of the protein to the asymmetric unit dimer interface. The channel in one monomer is contiguous to its counterpart in the other monomer thus forming a long and continuous hydrophobic tunnel (Fig. 6,7). As with AgamOBP1 crystal structure, we observed a continuous electron density in the course of this tunnel during the refinement procedure of AaegOBP1. It is worth mentioning that AaegOBP1 samples were delipidated [16] to remove possible ligand trapped in the binding pocket during expression and purification of protein samples. In addition, AaegOBP1 crystals were dissolved, extracted and analyzed by GC-MS, but we did not identify any low molecular weight ligand in these samples. Attempts to model the tunnel with oleic acid density led to a poor adjustment, whereas a PEG molecule showed a reasonable fit (Fig. 6,7). Even so, PEG in AaegOBP1 crystals could be modeled only to a stretch of 55 atoms in an ordered fashion as compared to 83 atoms in the AgamOBP1 structure [20]. This is equivalent to 61 atoms in AgamOBP1 given the differences in tunnel volumes. The calculated tunnel volume in AgamOBP1 is ca. 8.5% larger than in AaegOBP1, which may be related to difference in ligand specificity between the two proteins or merely an artifact from crystal packing. It is likely that mosquito OBPs exist in monomer-dimer equilibrium, with isolated dimers slowly converting to monomers [3]. Intriguingly, crystal packing interface analysis shows that the largest interface areas are the ones between the two monomers in the position they occur in both AaegOBP1 and AgamOBP1, and these two interfaces involve the same residues in the two proteins.

### Conclusion

We have determined the three-dimensional structure of AaegOBP1 and observed structural features strikingly similar to those previously described for another mosquito OBP, AgamOBP1. Contrary to what has been reported for a hymenopteran OBP [21], we did not find evidence for a domain-swapped dimer in this dipteran OBP. The C-terminal loops in both units of AaegOBP1 crystallographic dimer form “lids” that cover the binding pockets. As with another dipteran OBP [20], it is highly likely that lowering pH disrupts the hydrogen bonds that hold the C-terminal loop as a cover for the binding cavity. These findings are in line with the observed loss of binding affinity at low pH and a pH-mediated conformational change that retains the overall secondary structure while modifying the three-dimensional structure of the protein. Given the remarkable diversity of insects and their physiological systems, it would not be entirely surprising that hymenopteran olfactory proteins and dipteran, particularly mosquito OBPs, have different modes of action. Indeed, OBP from insect in a separate order (Lepidoptera) have a completely distinct mode of action, which has been well-documented in the literature [7], [8], [10], [12], [15], [16], [18], [19]. Moth OBPs, which are longer than dipteran and hymenopteran OBPs, have an extended C-terminus that forms an additional α-helix at low pH and competes with the ligand for the binding cavity. The structure of AaegOBP1 was determined with samples of a functional recombinant protein identical to the isolated protein, thus, supporting that our findings are physiologically relevant.

## Material and Methods

### Protein Expression and Purification

Total RNA was extracted from 1,000 antennae of female *A. aegypti* using TRIzol Reagent (Invitrogen, Carlsbad, CA). cDNA was synthesized by using SMART RACE cDNA Amplification Kit (Clontech, Mountain View, CA). Following primers were designed for preparation of the insert cDNA fragment: fAaegOBP1-KpnI, 5′-GCGGGGTACCCGACGTTACTCCGCGGCGTG-3′; rAaegOBP1-BamHI, 5′-GCGCGGATCCTTAAATCAGGAAGTAATGC-3′. *PfuUltra* Hotstart DNA polymerase (Stratagene, La Jolla, CA) was used as DNA polymerase. After heating at 95°C for 2 min, 30 cycles of PCR stepwise program (95°C for 30 s, 40°C for 30 s, and 72°C for 1 min) were carried out and subsequently heated at 72°C for 10 min. The amplified insert was gel-purified by QIAquick PCR Purification Kit (Qiagen, Valencia CA), and ligated into *Eco*RV recognition site of pBluescript II SK (+) (Stratagene) by using of T4 DNA ligase (New England Biolabs, Ipswich, MA). The insert cDNA fragment was verified by DNA sequencing at an automated sequencing facility (Davis Sequencing, Davis, CA).

Construction of the bacterial expression vector was carried out by the method described previously [3]. In brief, the selected vector with correct DNA sequence was digested with *Kpn* I (New England Biolabs), blunted by T4 DNA polymerase (New England Biolabs) with dNTP, and digested with *Bam* HI (New England Biolabs). The insert DNA was gel-purified by QIAquick Gel Extraction Kit (Qiagen), ligated into pET-22b(+) vector (Novagen, Gibbstown, NJ), and digested with *Msc* I (New England Biolabs) and *Bam* HI. This expression vector generates AaegOBP1 with identical amino acid sequence as the native protein [7] without truncation or any additional amino acid residues.

AaegOBP1 was expressed in LB medium with transformed BL21(DE3) cells, according to a protocol for perisplasmic expression of OBPs [7]. Proteins in the periplasmic fraction were extracted with 10 mM Tris**·**HCl, pH 8 by three cycles of freeze-and-thaw [8] and centrifuging at 16,000 ×*g* to remove debris. The supernatant was loaded on a Hiprep™ DEAE 16/10 column (GE Healthcare Bio-Sciences, Piscataway, NJ). All separations by ion-exchange chromatography were done with a linear gradient of 0–500 mM NaCl in 10 mM Tris·HCl, pH 8. Fractions containing the target protein were further purified on a 20-ml Q-Sepharose Hiprep™ 16/10 column (GE Healthcare Bio-Sciences) and, subsequently, on a Mono-Q HR 10/10 column (GE Healthcare Bio-Sciences). OBP fractions were concentrated by using Centriprep-10 (Millipore, Billerica, MA) and loaded on a Superdex-75 26/60 gel-filtration column (GE Healthcare) pre-equilibrated with 150 mM NaCl and 20 mM Tris**·**HCl, pH 8. Highly purified protein fractions were concentrated by Centricon-10, desalted on four 5-ml HiTrap desalting columns (GE Healthcare Bio-Sciences) in tandem with water as mobile phase, and analyzed by LC-ESI/MS (see below). The purest fractions were combined and delipidated following a previous protocol [16], with small modifications. Hydroxyalkoxypropyl-dextran type VI resin (Sigma, St. Louis, MO) (1 g) was suspended in HPLC grade methanol (20 ml), transferred to a glass column (i.d., 8.5 mm) with a stopper, washed with methanol (60 ml), and then equilibrated with 50 mM citric acid buffer, pH 4.5, after washing with 60 ml of this buffer. The content of the column was transferred to a 15 ml Falcon tube. Pure AaegOBP1 fractions (2–3 mg per delipidation batch) were dissolved in 50 mM citric acid, pH 4.5, mixed with the equilibrated resin, and incubated for 1 h at room temperature in a High Speed Rotating Extractor (RT50, Taitec, Tokyo, Japan) at 50 r/min. Then, the mixture was transferred to the glass column, AaegOBP1 was eluted with citric acid buffer, and analyzed by LC-ESI/MS. The purest fractions were desalted on a 5 ml HiTrap column (GE Healthcare, Bio-Sciences), analyzed by LC-ESI/MS, and the highest purity fractions (>99%) were used for crystallization and other analyses.

### Analytical Procedures

CD spectra were recorded with a Jasco J-810 spectropolarimeter (Easton, MD) with AaegOBP1 at 0.2 mg/ml (far-UV CD) and 1.6 mg/ml (near-UV CD), either in 20 mM ammonium acetate, pH 7, or in 20 mM sodium acetate, pH 5.5. LC-ESI/MS was performed with a LCMS-2010 (Shimadzu, Columbia, MD). High pressure liquid chromatography (HPLC) separations were carried out on a ZorbaxCB C8 column (150×2.1 mm; 5 µm; Agilent Technologies, Santa Clara, CA) with a gradient of water and acetonitrile plus 2% acetic acid as a modifier. The concentrations of the recombinant proteins were measured by UV radiation at 280 nm in 20 mM sodium phosphate, pH 6.5, and 6 M guanidine-HCl by using the theoretical extinction coefficients calculated with EXPASY software (http://us.expasy.org/tools/protparam.html).

### Crystallization, Diffraction Data Collection and Processing

Crystals were obtained by the hanging drop vapor diffusion method. Initial crystallization trials were prepared using polyethylene glycol 8000 (PEG) and Tris-HCl buffer. Suitable crystals were grown at 18°C in drops of 2 µL, made with equal volumes of protein at 50 µg/µL and reservoir solutions, the latter containing 30% (V/V) PEG 8000, 250 mM MgCl_2_ and 50 mM Tris–HCl, pH 8.5. Crystals were mounted in a nylon loop (Hampton Research, Aliso Viejo, CA) and flash frozen in a nitrogen gas stream at 100 K. Diffraction data were collected from one selected crystal at beam line X-12B of the Brookhaven National Laboratory with the wavelength set to 0.9000 Å and 1° oscillation for each frame (total 360 frames). The data were processed with *xds* and scaled with *xscale* from the *XDS* program suite [35]. AaegOBP1 crystallized in space group *P2_1_*, with unit cell parameters *a* = 34.37 Å, *b* = 47.93 Å, *c* = 69.12 Å and *β* = 96.50°. The statistics for data collection are summarized on Table 1.

### Structure Determination, Refinement and Analyses

The phase problem was solved by molecular replacement with the program *PHASER* (McCoy et al, 2005) using as search model one monomer of the structure of the OBP from *A. gambiae*, AgamOBP1 (PDB 2ERB; [20]), edited with the chainsaw program [36]. Refinement was carried out with *REFMAC5* [37]. Model building was performed interactively with the program *COOT* [38], which was used to gradually add water molecules as well as for several validation analyses. Other validation programs used were PROCHECK [27] and WHATCHECK [39]; some final refinement statistics are shown in Table 1. All pictures were created with PyMOL [40]. Structural superpositions were performed with either the program lsqkab (CCP4, 1994) or the Dalilite server (http://www.ebi.ac.uk/Tools/dalilite/index.html; [41]). Interface analyses were performed with the PISA Server (http://www.ebi.ac.uk/msd-srv/prot_int/pistart.html; [42]) while binding site volume calculations with the CASTp program [43]. The coordinates and structure factors have been deposited in the Protein Data Bank (3K1E).
